# Supplementary material for: Iron-sensing and redox properties of the hemerythrin-like domains of Arabidopsis BRUTUS and BRUTUS-LIKE2 proteins
Source: Nat Commun. 2025 Apr 24;16:3865. doi: 10.1038/s41467-025-58853-9 (PMC12022348; doi:10.1038/s41467-025-58853-9)
Supplement: Supplementary file 2 — Description of Additional Supplementary Files [file 41467_2025_58853_MOESM2_ESM.pdf]

Description of Additional Supplementary Files

Supplementary Data 1. SAXS experimental data and analysis.
