# Supplementary material for: Iron-sensing and redox properties of the hemerythrin-like domains of Arabidopsis BRUTUS and BRUTUS-LIKE2 proteins
Source: Nat Commun. 2025 Apr 24;16:3865. doi: 10.1038/s41467-025-58853-9 (PMC12022348; doi:10.1038/s41467-025-58853-9)
Supplement: Supplementary file 5 — Transparent Peer Review file [file 41467_2025_58853_MOESM5_ESM.pdf]

nature portfolio

Peer Review File

Iron-sensing and redox properties of the hemerythrin-like domains of Arabidopsis BRUTUS and BRUTUS-LIKE2 proteins

Corresponding Author: Dr Janneke Balk

This manuscript has been previously reviewed at another journal. This document only contains information relating to versions considered at Nature Communications.

This file contains all reviewer reports in order by version, followed by all author rebuttals in order by version.

A version of this paper was originally rejected for publication by Nature Communications, however that decision was reconsidered after appeal by the authors.

Version 1:

Reviewer comments:

Reviewer #1

(Remarks to the Author)
In this manuscript, the authors focused on the Hr-like domains of Arabidopsis BTS and BTSL2 proteins to investigate their iron-binding properties and redox activities in hopes of understanding the mechanism of conserved E3 ligase BTS proteins negatively mediating iron uptake via sensing iron levels and oxidative stress. By using the purified recombinant proteins of BTS-N, BTSL2-N, and their relevant variants, the authors did rigorous spectroscopic studies, in combination with preliminary structural analysis, to characterize the Hr-like domains of BTS/L biochemically and biophysically, and to further propose a working model for these E3s.

I do appreciate the characterization part which is thorough and intensive, however, except for the characterization, the presented study in current version seems lack of biology picture/significance as a whole, and more suitable for a journal specialized in biochemistry and/or chemistry rather than nature communication. To strengthen the biological significance of this study, the authors need to address, on top of their findings, the following questions, some of which are closely related to the open questions mentioned in the discussion section.

1. The authors demonstrated the binding dynamics and redox activities of diiron centers within the Hr-like domains of BTSL2 and BTS. Given these properties, when cellular iron status conditional on O2 or oxidative stress is sensed via the Hr-like domains, in other words, when diiron centers of Hr-like domains in different binding and redox states, what would happen to the substrate levels of these Hr-E3s, the substrate-E3s recognitions, or the downstream Fe uptake gene expressions? Although the authors have speculations regarding the E3 ligase activation upon iron and O2 sensing, some experimental data regarding this point, either in vitro or in vivo, would help establish the biological consequence/significance of their findings.

2. Triple Hr-like domains in tandem is such a unique feature of BTS E3s. Do these 3 Hr-like domains have crosstalk or interplay when sensing the iron and O2 or oxidative stress? Does the overall packing of 3 Hr-like domains contribute to the stability and thereby the activation of the BTS E3s? In particular, when compared with the Hr-E3s containing only one Hr domain such as FBXL5, do the BTS E3s have any uniqueness in iron and O2 sensing which is conferred by the triple Hr-like domains in tandem? The authors may consider using constructs containing single or double Hr-like domains to do spectroscopic and structural analyses. Experimental data addressing the interplay between or distinctive properties of the triple Hr-like domains would advance our understanding of Hr-like domain and its role in Hr-E3s, and thus add biological significance to this study.

Reviewer #2

(Remarks to the Author)
Summary: This is an excellent manuscript overall and I recommend that it be accepted for publication. The study is carefully done and it breaks new ground in understanding how these hemerythrin-like diiron found in E3 ligases function in sensing Fe and oxidative stress (OS) in plant cells. It's a novel system that will be of interest to the readers of Nature Communications. I'm only aware of the FBXL7 system and the "BRUTUS" proteins provide a second comparative example of these Fe sensing proteins. The senior authors are well-established researchers world-wide and are known for their careful and thorough work.

That being said, I do have some minor critical that should be considered and addressed prior to accepting the manuscript for publication in Nature Communications.
1. Hemerythrin is misspelled in the title.
2. One of the objectives of the study was to figure out why there are three Hr-like domains in these proteins, but I don't think they figured this out. I understand it would be a major endeavor to insert mutant proteins (with 1, 2, or 3 centers removed) into Arabidopsis to see the effect on iron homeostasis. I suspect there is cooperative assembly/disassembly of these centers that make the protein more sensitive to changes in Fe and OS than would otherwise be the case. This might be mentioned as a working hypothesis.
3. The EPR signal for Cu(EDTA) will saturate using a microwave power of 3.17 mW at 10 K. The authors should redo the measurement under nonsaturating conditions. I'm afraid that this will make the integrated intensity of the mixed-valent and g = 4.3 signals significantly lower (unless the signals from the protein are also saturated). Also, my impression of quantifying a g= 4.3 signal is that one needs to know the zero-field splitting since this signal is emanating from only the middle doublet of the S = 5/2 spin manifold. Thus, if all three doublet levels were equally populated, one would need to multiply the observed intensity by 3. Also, there is a correction (discovered by Aasa and Vangard) involving the g-values; the authors should include these factors in accurately quantifying these intensities.
4. Related to this, one of the major missing issues in the manuscript is a discussion of the presence or absence of the oxo or hydroxo group in these centers. The presence of this group could be established for the mixed-valence state since the irons are antiferromagnetically coupled. This would be helped by trying to maximize the intensity better. Have they tried adding exactly 1 electron equivalent of dithionite (or ascorbate) per center to the fully-oxidized protein, and then examining the EPR? For these centers, it would be unusual for get full spin quantitation, but obtaining even 0.1 spins per center would be meaningful and similar to what others have seen. Then the authors could argue that an oxo bridge must also be present in the fully oxidized state. I'm not certain how they could establish the bridge in the fully reduced state…perhaps Mossbauer depending on whether the center would be antiferromagnetically coupled.
5. Speaking of Mossbauer, it would be interesting to examine the reduced enzyme prepared from the apo protein under anaerobic conditions, such that there might be two FeII ions bound but not a bridging oxo or OH group as would likely be found under aerobic conditions. I'm wondering whether the bridging OH group controls whether the FeII ions reversibly dissociate.
6. The Figure 1 legend mentions a question mark in the figure but I couldn't find it.
7. Page 5: states that the metal centres are "close to one another" but 21 A apart seems pretty far apart. Are the authors suggesting some cluster-cluster interactions? Or electron transfer connectivity? At this distance, electron transfer from one center to the next would likely be very slow.
8. Page 11: The EPR spectra are not as definitive as suggested in ruling-out Fe/S clusters. The EPR of the reduced protein lacks a g = 1.94 signal, but not all Fe/S clusters are reducible as their potentials might be very low. I agree that Fe/S clusters are unlikely, but perhaps this is more compelling due to the lack of conserved Cys residues. Mossbauer spectroscopy could have been more definitive in this regard.
9. Page 14: typo iron not irn
10. I was concerned about the concept of the clusters being "locked" in to the FeIII-O-FeIII state at high Fe and high OS (page 16). If these proteins were dynamically sensing Fe and OS, wouldn't it be better for the activation to be irreversible? … for example, in the event of the cell reestablishing a reducing environment? In that case, you'd want the FeII to dissociate from the centers to turn off degradation of the transcription factor, right?
11. Page 19: line 473… E. coli cells.
12. Page 19, line 478 (manufacturer and model of sonicator). Also, model of Thermo Scientific ICP-MS.
13. The mechanism by which these proteins sense Fe and OS is very nice but the authors ought to compare and contrast to the function of the FBXL5 system more. Are there any major differences in how these proteins function?

All things considered, this is an exciting and well-done study that should be published after these minor revisions. Sincerely, Reviewer #2

Reviewer #3

(Remarks to the Author)
The manuscript entitled "Iron and redox sensing in plants: properties of the hemerythin-like domains of Arabidopsis BRUTUS and BRUTUS-LIKE2 proteins" by Pullin, Balk and coworkers describes the iron-binding properties and redox activities of the Hr-like domain at the N-terminus and proposes a model for its sensor mechanism.

I was asked to particularly assess the SAXS part of the study, thus this review focusses exclusively on the SAXS results and analysis.

The experimental procedures for SEC-SAXS are adequately described and the SAXS data as presented in Fig. 2c seem

solid. However, there are several major problems with data analysis and interpretation.

Major comments:

In my opinion the SAXS data are overinterpreted and exaggerated. SAXS data cannot be used to “validate” an AlphaFold2 model as stated in the introduction (l.59-60) and as title for results section #2 (l.121). An ab initio SAXS model as shown in Fig.2b is always ambiguous, i.e. many different protein models would result in the same 3D envelope. The proposed BTSL2 Hr-like domain arrangement is certainly in agreement with the SAXS data, but it is not a validation.

A CRYSOL analysis would be more suitable than fitting a model into a low-res “blob” to compare experimental SAXS data to the AF2 model, but even this would not be a “validation”, which is overstated.

Several aspects of SAXS data analysis are simply wrong. Fig. S1C shows a distance distribution plot and not a Porod plot (which would be I(s)*s4 vs. s). In addition, this distance distribution plot does not show a shoulder indicating a multi-domain structure as stated by the authors in line 146-147. The minor feature in the P(r) curve around 90A is caused by not adjusting the alpha-value and does not indicate any multi-domain properties.

Publication guidelines for SAXS data have published in 2017 (Trewhella et al, PMID: 28876235), which are commonly used in the field and should be used in any publication with significant SAXS content.

Overall, there are significant problems with SAXS data analysis and interpretation and I don’t believe that the SAXS part (even if analysed correctly) will add any significant insight to the story.

Reviewer #4

(Remarks to the Author)
I evaluated the SAXS data, which was of good quality. The Guinier plot showed no protein aggregation, and there were no problems with interpreting the Kratky plot. However, I would like to request the following issues.

1: The scattering curve of the experimental data should be compared with the curve calculated from the BTSL2-N AF2 atomic model using the CRYSOL program in the ATSAS package.

2: The authors should prepare the table for SAXS experiment and analysis according to the publication guideline for bioSAXS (Jill Trewhella et la Acta Cryst D (2017) 73, 710-728).

3: I could not understand whether the scattering intensity was normalized on the absolute scale using the scattering intensity of water.

Version 2:

Reviewer comments:

Reviewer #1

(Remarks to the Author)
The authors did address most of my comments in the initial review with analysis, discussion, and additional data. I am therefore now satisfied with this revised version of manuscript for publication. I thank the authors for their thorough and thoughtful responses.

Reviewer #2

(Remarks to the Author)
The authors have responded appropriately to my comment. I recommend the paper for publication.

Reviewer #3

(Remarks to the Author)
The authors have adequately addressed all my concerns regarding SAXS analysis.

Reviewer #4

(Remarks to the Author)
The authors have adequately addressed my concerns. I have no further comments.

Open Access This Peer Review File is licensed under a Creative Commons Attribution 4.0 International License, which permits use, sharing, adaptation, distribution and reproduction in any medium or format, as long as you give appropriate credit to the original author(s) and the source, provide a link to the Creative Commons license, and indicate if changes were made.
In cases where reviewers are anonymous, credit should be given to 'Anonymous Referee' and the source.
The images or other third party material in this Peer Review File are included in the article’s Creative Commons license, unless indicated otherwise in a credit line to the material. If material is not included in the article’s Creative Commons license and your intended use is not permitted by statutory regulation or exceeds the permitted use, you will need to obtain permission directly from the copyright holder.
To view a copy of this license, visit https://creativecommons.org/licenses/by/4.0/

Response to reviewers' comments

Reviewer #1:
In this manuscript, the authors focused on the Hr-like domains of Arabidopsis BTS and BTSL2 proteins to investigate their iron-binding properties and redox activities in hopes of understanding the mechanism of conserved E3 ligase BTS proteins negatively mediating iron uptake via sensing iron levels and oxidative stress. By using the purified recombinant proteins of BTS-N, BTSL2-N, and their relevant variants, the authors did rigorous spectroscopic studies, in combination with preliminary structural analysis, to characterize the Hr-like domains of BTS/L biochemically and biophysically, and to further propose a working model for these E3s.

I do appreciate the characterization part which is thorough and intensive, however, except for the characterization, the presented study in current version seems lack of biology picture/significance as a whole, and more suitable for a journal specialized in biochemistry and/or chemistry rather than nature communication. To strengthen the biological significance of this study, the authors need to address, on top of their findings, the following questions, some of which are closely related to the open questions mentioned in the discussion section.

- We have strengthened the biological significance (see below), but would also like to emphasize that biochemical data of the BTS/L proteins have been sorely lacking, despite several high profile papers (Nature Comms, Nature Plants, PNAS, Plant Cell) on the biological function of these proteins since 2013.

1. The authors demonstrated the binding dynamics and redox activities of diiron centers within the Hr-like domains of BTSL2 and BTS. Given these properties, when cellular iron status conditional on O2 or oxidative stress is sensed via the Hr-like domains, in other words, when diiron centers of Hr-like domains in different binding and redox states, what would happen to the substrate levels of these Hr-E3s, the substrate-E3s recognitions, or the downstream Fe uptake gene expressions? Although the authors have speculations regarding the E3 ligase activation upon iron and O2 sensing, some experimental data regarding this point, either in vitro or in vivo, would help establish the biological consequence/significance of their findings.

- We agree that experimental data on the signal transduction in response to iron binding and redox changes is important to strengthen the model. Ideally, an experimental set up with recombinant full-length BTS and its substrate protein(s) would enable us to study this in vitro. So far, we have only managed to produce small amounts of full-length protein using a wheat-germ expression system or, even smaller amounts, using transient expression in *Nicotiana benthamiana* leaves. Unfortunately, the E3 ligase activity (as measured by autoubiquitination) was lost during purification, and the small amounts of protein limit the scope for optimization. Instead, we have focussed on characterizing the iron binding and redox properties of a BTS variant protein with a mutation in the Hr-like 1 subdomain. We recently identified the so-called *dgl* mutation in pea (Harrington *et al* 2024 Plant J), of which the mutant phenotype had already been extensively studied over the past decades. In particular, a paper from 2013 showed that iron uptake genes downstream of BTS and its substrates are constitutively expressed (Garcia et al 2013 Planta), indicating diminished activity of BTS in *dgl* plants. Our new data show that the BTS^{dgl}-N protein binds 4 irons compared to 6 in wild-type BTS, from which we conclude that iron binding in the H1 site is completely abolished, at least in recombinant protein. We also found that the *dgl* variant could not be fully reoxidized, either because iron is cooperatively lost from the other two diiron sites, or because intra-molecular electron transfer is impaired. The data are added in a new Figure 8.

2. Triple Hr-like domains in tandem is such a unique feature of BTS E3s. Do these 3 Hr-like domains have crosstalk or interplay when sensing the iron and O2 or oxidative stress? Does the overall packing of 3 Hr-like domains contribute to the stability and thereby the activation of the BTS E3s? In particular, when compared with the Hr-E3s containing only one Hr domain

such as FBXL5, do the BTS E3s have any uniqueness in iron and O2 sensing which is conferred by the triple Hr-like domains in tandem? The authors may consider using constructs containing single or double Hr-like domains to do spectroscopic and structural analyses. Experimental data addressing the interplay between or distinctive properties of the triple Hr-like domains would advance our understanding of Hr-like domain and its role in Hr-E3s, and thus add biological significance to this study.

- The reviewer raises important questions, which are at least partially answered by our results. In brief, there is evidence for crosstalk because removal of one diiron site through mutagenesis alters iron binding to the other site in BTSL2 (Fig. 6), absorbance properties (Fig. 3) and affects the mixed valence EPR signals (Supplementary Fig. 4c). Similarly, lack of the diiron cofactor in the H1 site of BTS impaired reoxidation of the other two cofactors (new Fig. 8) and also affected the mixed valence signal in the EPR spectrum (new Supplementary Fig. 4b). The packing of the 3 Hr-like motifs is such that the domain as a whole is mostly inflexible (SAXS data, Supplementary Fig. 1b, Kratky analysis). Based on the AF2 model, the triple domain could undergo a hinging movement relative to the E3 ligase domain and possibly have an auto-inhibitory function. Indeed, this is consistent with in vivo data showing that removal of the triple Hr-like domain in BTS results in constitutive activity (Selote et al 2015 Plant Physiol). Comparing our data with those reported for FBXL5, the triple Hr-like domain binds iron more tightly during redox cycling, which is summarized in the Discussion. Thus, the molecular mechanism of BTS/L is likely to be different from that proposed for FBXL5.
  When we started the biochemical characterization of the BTS proteins more than 10 years ago, we expressed the individual Hr-like subdomains of Arabidopsis BTSL1 in *E. coli*. The first domain (H1) was difficult to express (Fig. R1a), which made sense when the AF2 model became available: H1 is stabilized by H2 and H3 through large interaction surfaces. Further characterization of H3 showed it binds 2 irons, in agreement with a UV-visible spectrum typical of diiron proteins when oxidized (Fig. R1b). However, we felt that studying the three Hr motifs together would be more relevant, also because phylogeny analysis showed that there must be biological importance of the triple Hr domain, given its conservation in BTS proteins throughout the green lineage (Fig. 1a). Rather than protein versions with one or two Hr-like domains, we have studied mutant versions lacking one or both diiron centres (Fig. 3, 6 and 8). We have also initiated work to generate plant lines in which individual iron-binding sites are mutated, but this takes time (see Reviewer #2, point 2) and would add a whole chapter of plant biology to the mostly biochemical and biophysical analyses.

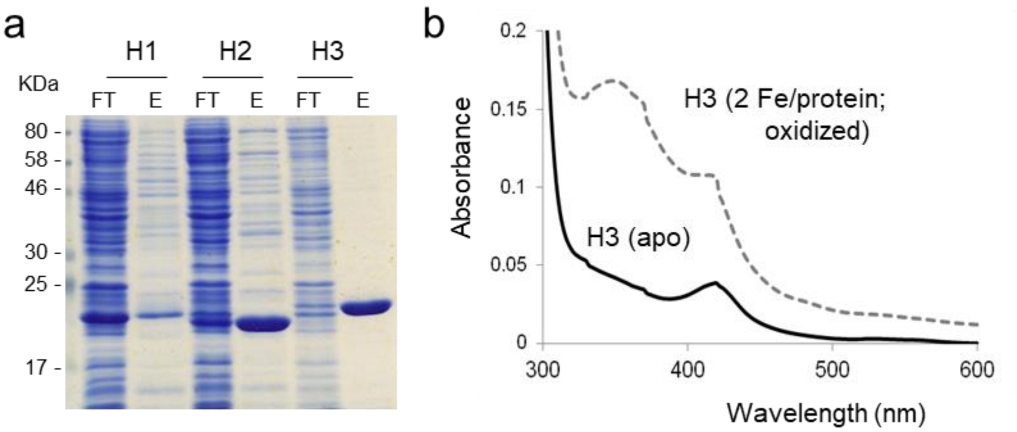

Figure R1. Expression and UV-visible spectra of individual Hr-like domains of Arabidopsis BTSL1.
a Purification of individual Hr-like subdomains (H1, H2 and H3) of Arabidopsis BTSL1 produced in *E. coli*. The proteins had an N-terminal His-tag and were purified by nickel NTA affinity chromatography. Flow through (FT) and eluted purified proteins (E) were loaded on an SDS-PAGE gel and stained with Coomassie. **b** UV-Visible spectra of: the BTSL1 H3 domain purified from semi-anaerobic *E. coli* cultures, containing 2 iron/protein in the oxidized

state (by exposure to air during purification); and the H3 domain purified from well-oxygenated *E. coli* cultures, which was predominantly in the apo form.

Reviewer #2:

Summary: This is an excellent manuscript overall and I recommend that it be accepted for publication. The study is carefully done and it breaks new ground in understanding how these hemerythrin-like diiron found in E3 ligases function in sensing Fe and oxidative stress (OS) in plant cells. It's a novel system that will be of interest to the readers of Nature Communications. I'm only aware of the FBXL7 system and the "BRUTUS" proteins provide a second comparative example of these Fe sensing proteins. The senior authors are well-established researchers world-wide and are known for their careful and thorough work.

That being said, I do have some minor critical that should be considered and addressed prior to accepting the manuscript for publication in Nature Communications.

1. Hemerythrin is misspelled in the title.

- Corrected.

2. One of the objectives of the study was to figure out why there are three Hr-like domains in these proteins, but I don't think they figured this out. I understand it would be a major endeavor to insert mutant proteins (with 1, 2, or 3 centers removed) into Arabidopsis to see the effect on iron homeostasis. I suspect there is cooperative assembly/disassembly of these centers that make the protein more sensitive to changes in Fe and OS than would otherwise be the case. This might be mentioned as a working hypothesis.

- We thank the reviewer for the suggestion. See also Reviewer #1, point 1. We have added new data (Fig. 8) on a mutant variant of BTS that lacks the diiron centre in the H1 subdomain. Previous phenotyping studies showed that iron homeostasis is greatly perturbed as a consequence of diminished BTS functionality (although BTS had not been characterized at the time, nor linked to the *dgl* mutation!). Interestingly, altered redox behaviour and EPR spectroscopy of the variant protein suggest cooperativity/inter-dependency between the diiron centres. We have added this as a working hypothesis to the Discussion (lines 465 – 479 in highlighted manuscript file).

3. The EPR signal for Cu(EDTA) will saturate using a microwave power of 3.17 mW at 10 K. The authors should redo the measurement under nonsaturating conditions. I'm afraid that this will make the integrated intensity of the mixed-valent and g = 4.3 signals significantly lower (unless the signals from the protein are also saturated). Also, my impression of quantifying a g= 4.3 signal is that one needs to know the zero-field splitting since this signal is emanating from only the middle doublet of the S = 5/2 spin manifold. Thus, if all three doublet levels were equally populated, one would need to multiply the observed intensity by 3. Also, there is a correction (discovered by Aasa and Vangard) involving the g-values; the authors should include these factors in accurately quantifying these intensities.

- Cu^{2+}[EDTA] will indeed saturate at such high microwave powers; quantification was carried out at P_{MW}= 3.17 x 10^{-3} mW where both the mixed valence and the copper standard would be under non-saturating conditions. Similarly, the g= 4.3 signals were measured at P_{MW}= 0.05 mW. We apologise for not giving full details of the method. To be certain of the quantification we have repeated these EPR measurements as well. Additional details of the quantification are now given in the Methods section and the power saturation curves for the Cu^{2+}[EDTA] standard and proteins have been added as Supplementary Fig. 10 and copied below:

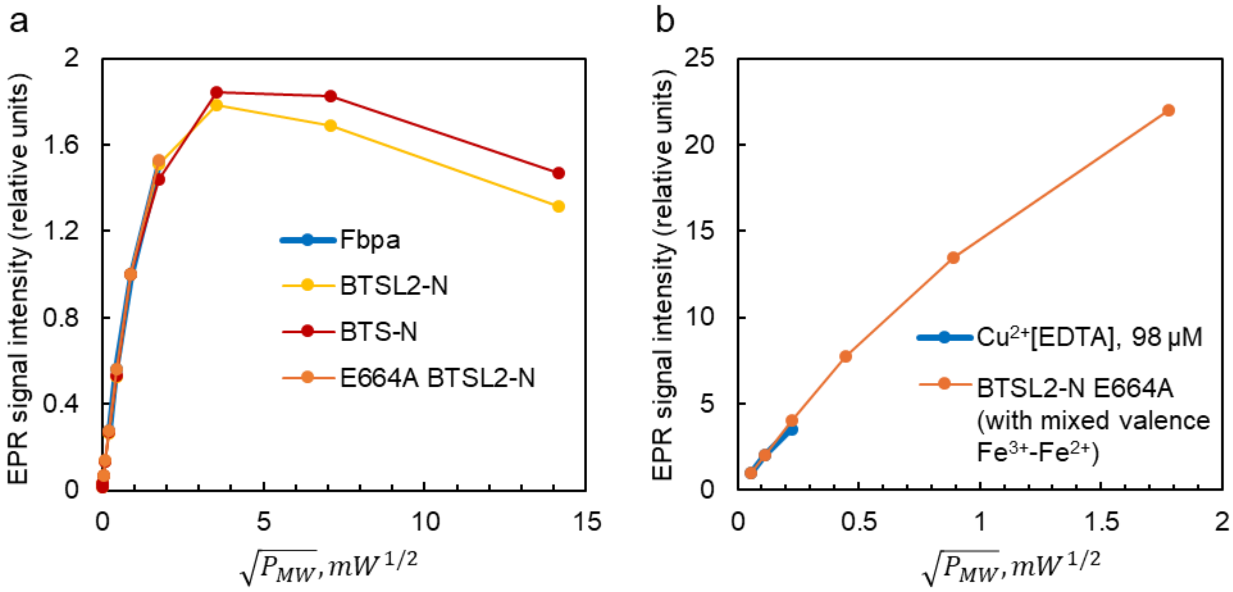

Supplementary Fig. 10. Power saturation curves for the EPR signals observed in BTS/L-N proteins. **a** Power saturation curves for the g = 4.3 signals of Fbpa (blue), BTSL2-N (yellow), BTS-N (red) and BTSL2-N E664A (orange). Each curve is normalised to an identical slope for the initial linear parts of the dependences (no saturation at low powers) with intensity 1.00 set for the microwave power of P_{MW} = 0.8 mW. **b** Power saturation curves for a 98 μM Cu^{2+}[EDTA] standard, and for the EPR signal from the mixed-valent state of the diiron centre in BTSL2-N E664A (527 μM). The two curves are normalised as in (a) with intensity 1.00 set for the microwave power of P_{MW} = 0.00317 mW.

4. Related to this, one of the major missing issues in the manuscript is a discussion of the presence or absence of the oxo or hydroxo group in these centers. The presence of this group could be established for the mixed-valence state since the irons are antiferromagnetically coupled. This would be helped by trying to maximize the intensity better. Have they tried adding exactly 1 electron equivalent of dithionite (or ascorbate) per center to the fully-oxidized protein, and then examining the EPR? For these centers, it would be unusual for get full spin quantitation, but obtaining even 0.1 spins per center would be meaningful and similar to what others have seen. Then the authors could argue that an oxo bridge must also be present in the fully oxidized state. I'm not certain how they could establish the bridge in the fully reduced state…perhaps Mossbauer depending on whether the center would be antiferromagnetically coupled.

- We performed anaerobic titrations of sodium dithionite with BTSL2-N or BTS-N to try and increase the signal intensity of the mixed-valence diiron centres. Of the two mixed valence signals in BTSL2-N, only one changed amplitude to double that of the as-isolated protein, peaking when enough dithionite was added to reduce 25% of the iron. However, the signal remains very small as a percentage of total iron. For BTS-N, no changes of the mixed valence signal were observed in response to dithionite titration. We have added a sentence to the Results section to report on the (negative) results, including new Supplemental figure 4a and methods description in Supplementary Information.

5. Speaking of Mossbauer, it would be interesting to examine the reduced enzyme prepared from the apo protein under anaerobic conditions, such that there might be two FeII ions bound but not a bridging oxo or OH group as would likely be found under aerobic conditions. I'm wondering whether the bridging OH group controls whether the FeII ions reversibly dissociate.

- We agree that Mossbauer studies would be informative to address this point, as well as other questions such as whether the diiron centre is penta or hexa coordinated, and pinpointing which iron(s) are dynamic. However, in our hands the purified BTS/L

proteins are unstable in the apo form, and formed a jelly at higher concentrations that are required for Mossbauer spectroscopy, issues that need to be resolved first.

6. The Figure 1 legend mentions a question mark in the figure but I couldn't find it.

- The contrast has been improved. We have also updated the phylogenetic tree to include sequences for gymnosperms, which we could not find initially. They are quite diverged but do have the triple Hr-like domain.

7. Page 5: states that the metal centres are "close to one another" but 21 A apart seems pretty far apart. Are the authors suggesting some cluster-cluster interactions? Or electron transfer connectivity? At this distance, electron transfer from one center to the next would likely be very slow.

- In isolation, the diiron centres would indeed to be too far apart for rapid electron transfer. However, they may be connected by electron transfer through Trp and Tyr residues, as suggested by the AF2 model. In fact, those residues are conserved and a future target of mutagenesis studies.

8. Page 11: The EPR spectra are not as definitive as suggested in ruling-out Fe/S clusters. The EPR of the reduced protein lacks a g = 1.94 signal, but not all Fe/S clusters are reducible as their potentials might be very low. I agree that Fe/S clusters are unlikely, but perhaps this is more compelling due to the lack of conserved Cys residues. Mossbauer spectroscopy could have been more definitive in this regard.

- There are some conserved Cys residues in the Hr-like subdomains, but not in patterns that are typical for Fe-S clusters. So they could be disulphide bridges or an usual Fe-S cluster. For this reason, we'd rather not use the conserved Cys residues as an argument, but point to the EPR and CD data instead, which together provide strong evidence (UV-vis and CD show redox reversibility, and EPR a lack of any Fe-S signals in either oxidised or reduced spectra). We have re-written this part of the manuscript (lines 271 – 273, highlighted version) to better explain why we think the evidence is clear. Regarding Mossbauer analysis, see point 5.

9. Page 14: typo iron not irn

- Corrected.

10. I was concerned about the concept of the clusters being "locked" in to the FeIII-O-FeIII state at high Fe and high OS (page 16). If these proteins were dynamically sensing Fe and OS, wouldn't it be better for the activation to be irreversible? …for example, in the event of the cell reestablishing a reducing environment? In that case, you'd want the FeII to dissociate from the centers to turn off degradation of the transcription factor, right?

- We assume the reviewer meant 'reversible'? We have changed the wording to "shifts the protein into its active conformation" so as not to imply that this is an irreversible process. Indeed, when the cell is no longer under iron/ROS stress, then the diiron centres would be reduced and iron could dissociate.

11. Page 19: line 473… E. coli cells.

- Corrected.

12. Page 19, line 478 (manufacturer and model of sonicator). Also, model of Thermo Scientific ICP-MS.

- Information has been added.

13. The mechanism by which these proteins sense Fe and OS is very nice but the authors ought to compare and contrast to the function of the FBXL5 system more. Are there any major differences in how these proteins function?

- ➢ We have expanded the paragraph in the Discussion that addresses differences in the iron binding and redox properties, to also discuss functional differences as a consequence of having the triple Hr-like domain in BTS rather than a single domain in FBXL5. Mainly, the triple domain could display cooperative iron dis/assembly (see point 2) and it could form an auto-inhibitory domain (see Reviewer #1, point 2).

All things considered, this is an exciting and well-done study that should be published after these minor revisions.

Reviewer #3:
The manuscript entitled "Iron and redox sensing in plants: properties of the hemerythin-like domains of Arabidopsis BRUTUS and BRUTUS-LIKE2 proteins" by Pullin, Balk and coworkers describes the iron-binding properties and redox activities of the Hr-like domain at the N-terminus and proposes a model for its sensor mechanism.
I was asked to particularly assess the SAXS part of the study, thus this review focusses exclusively on the SAXS results and analysis.

The experimental procedures for SEC-SAXS are adequately described and the SAXS data as presented in Fig. 2c seem solid. However, there are several major problems with data analysis and interpretation.

Major comments:
1. In my opinion the SAXS data are overinterpreted and exaggerated. SAXS data cannot be used to "validate" an AlphaFold2 model as stated in the introduction (l.59-60) and as title for results section #2 (l.121). An ab initio SAXS model as shown in Fig.2b is always ambiguous, i.e. many different protein models would result in the same 3D envelope. The proposed BTSL2 Hr-like domain arrangement is certainly in agreement with the SAXS data, but it is not a validation.
A CRYSOL analysis would be more suitable than fitting a model into a low-res "blob" to compare experimental SAXS data to the AF2 model, but even this would not be a "validation", which is overstated.

- ➢ We thank the reviewer for their insightful comments here. We agree the verb "validate" is not correct and we have changed the wording in all three instances. CRYSOL analysis has been carried out and the χ^2 was 1.008. This has been added to the text, lines 146 – 147 in highlighted version).

*2.Several aspects of SAXS data analysis are simply wrong. Fig. S1C shows a distance distribution plot and not a Porod plot (which would be I(s)*s4 vs. s). In addition, this distance distribution plot does not show a shoulder indicating a multi-domain structure as stated by the authors in line 146-147. The minor feature in the P(r) curve around 90A is caused by not adjusting the alpha-value and does not indicate any multi-domain properties.*

- ➢ We apologize for the errors and have corrected them. The legend for Fig. S1c now reads "P(*r*) versus *r* profile" following the example in Trewhella et al 2017. For the graph shown, alpha was 0.007; increasing this value does indeed smooth out the feature around 90 Ångstrom. We have replaced the P(r) curve and deleted the sentence "The presence of a shoulder in the P(r) distribution (Figure S1C) is indicative of the multi-domain structure of BTSL2-N".

3. Publication guidelines for SAXS data have published in 2017 (Trewhella et al, PMID: 28876235), which are commonly used in the field and should be used in any publication with significant SAXS content.

- ➢ We used this paper, and the 2023 update, extensively and have cited it (Refs 51-52).

4. Overall, there are significant problems with SAXS data analysis and interpretation and I don't believe that the SAXS part (even if analysed correctly) will add any significant insight to the story.

- The SAXS data are important because they indicate that the three Hr-like domains are tightly packed in a rigid "triple motif domain", which is very different from the mammalian FBXL5 protein (featuring only one Hr motif). Both Reviewer #1 (point 2) and Reviewer #2 (point 13) highlight this unique feature. Moreover, and we present evidence that there is inter-dependency/cooperativity between the Hr motifs, indicating that the domain structure is highly relevant for the regulatory mechanism. We have stressed this more throughout the manuscript.

Reviewer #4:

I evaluated the SAXS data, which was of good quality. The Guinier plot showed no protein aggregation, and there were no problems with interpreting the Kratky plot. However, I would like to request the following issues.

1: The scattering curve of the experimental data should be compared with the curve calculated from the BTSL2-N AF2 atomic model using the CRYSOL program in the ATSAS package.

- See also Reviewer #3, point 2. The analysis was carried out but not mentioned. We have now added it to the text, lines 146 – 147 in highlighted version)..

2: The authors should prepare the table for SAXS experiment and analysis according to the publication guideline for bioSAXS (Jill Trewhella et la Acta Cryst D (2017) 73, 710-728).

- Apologies, this table was prepared but inadvertently omitted from the submission. We have now included it in the Supplemental Data file (Table S2).

3: I could not understand whether the scattering intensity was normalized on the absolute scale using the scattering intensity of water.

- We apologise that this was not clear. It refers to a standard procedure. First, the empty capillary was measured, then the capillary filled with water, and the intensity was normalized to the water scattering of 0.0163 cm^{-1}. This will give scattering in an absolute scale. We have added this information to the Methods (lines 559 – 560 in highligted version).
